# Narcotics Anonymous members in recovery from methamphetamine use disorder

**DOI:** 10.1111/ajad.13362

**Published:** 2022-11-25

**Authors:** Marc Galanter, William L. White, Brooke Hunter

**Affiliations:** ^1^ Department of Psychiatry New York University School of Medicine New York New York USA; ^2^ Research Division Chestnut Health Systems Punta Gorda Florida USA; ^3^ Research Division Chestnut Health Systems Normal Illinois USA

## Abstract

**Background and Objectives:**

Methamphetamine use disorder (MUD) is a major public health problem, but there are no evidence‐based, best‐practice, pharmacologic, or behavioral treatments for it. Narcotics Anonymous (NA) may provide an option for referral for such patients.

**Methods:**

Two waves of surveys were sent to a sample of NA members to evaluate demographic, drug use, and NA‐related issues. Of 4445 responses received from US residents, 647 listed themselves as abstinent from their worst drug problem, methamphetamine. Twelve possible sources of support were scored by these latter respondents for how important each was for their own recovery.

**Results:**

Methamphetamine respondents were longstanding NA members, with their first NA meeting 30.2 years ago, 84.3% having served as sponsors for other members, and with little current craving (0.65 out of 10). Although now abstinent for an average of 13.4 years, at some point over the course of the membership, 47.4% had experienced a relapse, for an average of 16.7 months. In a factor analysis of resources scored, 29.6% of the variance fell under NA social and 29.2% spiritual; and 11.8% under outside professional support.

**Discussion and Conclusions:**

NA served as a resource for supporting abstinence for some members with MUD. They scored social resources of NA support higher than both spiritual and outside institutional ones.

**Scientific Significance:**

NA can serve as a community‐based resource for MUD. Determining the nature of recovery that members with MUD have in NA can be useful for further research of socially grounded support for recovery in substance use disorders.

## INTRODUCTION

There are limited pharmacologic treatment options for methamphetamine (N‐methamphetamine) use disorder (MUD)^1^ and approaches for promoting recovery rely on nonpharmacologic, psychosocial care^2^, ^3^, ^4^, ^5^ because there are no Food and Drug Administration (FDA)‐approved medications for MUD, and most efforts for promoting recovery rely on nonpharmacologic, psychosocially based approaches. The Twelve Step‐based fellowship Narcotics Anonymous (NA) may serve as an option to aid some persons with MUD in achieving recovery from this disorder. Because of this, we studied a sample of NA members who reported methamphetamine as their principal drug problem.

Although methamphetamine can be used singly, it can also be injected intravenously in combination with heroin.^6^ Its clinical manifestations can be contrasted with those of the other major stimulant subject to misuse, cocaine, which has a slower onset of action and a longer duration of effect. Depending on these factors, the effects of the two may be comparable in onset and duration. Neurotoxic effects of methamphetamine occur from repeated use, resulting in neurocognitive defects, and persisting even after a period of abstinence.^7^

In 2012, federal legislation was passed to limit over‐the‐counter sales of ephedrine and pseudoephedrine because of their use in synthesizing methamphetamine in local US laboratories.^8^ Subsequent illicit importation of the drug from Mexico, however, has made large quantities available. Consequently, subsequent increased availability of higher purity methamphetamine has made medical consequences more prominent^9^; use of the drug has increased considerably in the United States in recent years. As seen in a report from the US Center for Disease Control and Prevention, among adults reporting methamphetamine use in 2018, 27.3% reported using it on more than 200 days, and 22.3% had injected it.^10^ Data from poison control centers show admission to healthcare facilities rose from 30.2% in 2000 to 47.8% in 2019.^11^ Methamphetamine‐related hospital admissions as a portion of all drug‐related admissions^10^ increased from 15.1% in 2008 to 23.6% in 2017. Increased attention to opioids in recent years, such as heroin and fentanyl, has predominated regarding the problem of drug overdoses, but by 2018, methamphetamine accounted for 11% of all reported overdose deaths.^8^

Despite the high prevalence of methamphetamine use in the United States and its notable contribution to overdose deaths, the availability of treatment for this major public health problem is limited. Numerous pharmacologic agents have been applied to address MUD in clinical trials in an attempt to reduce this drug's misuse, such as modafinil,^12^ topiramate,^13^ sertraline,^14^ and naltrexone.^15^ A reduction in craving has not been reported in a comprehensive literature review on clinical pharmacologic trials.^16^ The National Institute on Drug Abuse (NIDA) has pointed out that there are currently no medications “that prolong abstinence or reduce misuse” of the drug.^17^ With regard to psychosocial treatment, two uncontrolled studies have shown positive results, one on contingency management^18^ and another employing the Matrix model, a multimodal, manualized approach.^19^ NIDA points out that the “most effective” treatments for MUD are behavioral, such as cognitive behavioral and contingency management interventions.^17^

## METHOD

We established an agreement with the NA World Service office, located in Chatsworth, CA, for their membership office to email anonymous surveys to subscribers of the NA newsletter. Its purpose was to assess experience in the fellowship relative to recovery from substance use disorder (SUD). The initiative to study NA members who designated methamphetamine as their principal SUD was approved by the Institutional Review Board of the Chestnut Health System, with no consent required. Emails were sent to NA members stating that they could volunteer to respond to the anonymous survey if they chose to do so. Anonymous responses were analyzed by the authors for US respondents, with the files to be subsequently deleted to protect respondents' personal information.

COVID‐19 stay‐at‐home restrictions were first announced in New York, California, Illinois, and Washington States in March 2020, and lifted in most States by June 2021. The surveys prepared were sent in two waves, one in June 2020, and the second in March 2021. This allowed for assessing the impact of relative isolation during the stay‐at‐home period of COVID‐19 on the respondents' participation in NA meetings and when restrictions were being lifted. The use of two waves also allowed for limiting the amount of time the data solicited would be required for completion in a given sitting.

### Measures

In the first wave survey (June 2020), respondents were asked to score demographic, substance use, and recovery‐related items. Two types of meetings were distinguished: face‐to‐face (f/f), those held in the traditional fashion, with participants located in the same place, and virtual, those held on the internet, typically in the Zoom format. Craving for drugs or alcohol was assessed by response to a 0‐ to 10‐point visual analog scale, similar to one applied in previous surveys.^20^ Duration of abstinence was queried in the survey: “How many months has it been since you last used alcohol or drugs?”

In the second wave survey (June 2021), 12 potential sources of support for recovery were listed, both before and during the COVID‐19 period. These were based on the experience of two of the authors' past interviews with NA members and their published research.^20^ The sources of support were: *Other members* you got to know, *face‐to‐face* meetings, NA Basic Text and *NA literature*, *my sponsor*, your own *service work*, *God, NA Prayers, spiritual awakening*, and *meditation*. Three institutionally sanctioned items, outside the umbrella of NA, were also queried: a professional *therapist*, *medication* for psychological distress, and *attendance at a house of worship* (like a church). These labels are abbreviated in the text and tables as italicized above. In the second survey, respondents were asked to score each of these 12 items for how important they were for support for their own recovery. To ascertain respondents' relative reliance on a given source of support independent of the impact of COVID‐19, they were also asked to score items of support for abstinence before the COVID‐19 period: 0 for not important, 1 for slightly important, 2 for fairly, 3 for very, or 4 for extremely important.

A factor analysis was executed on the 12 sources of support for the period *Before COVID‐19* using alpha factoring with an equamax rotation and suppression threshold of 0.30. The *Before COVID‐19* time period was further analyzed by dichotomizing Likert responses to compare respondents who indicated a score of 1–4 (important, at least to some degree) as a source of support versus those responses indicating a score of zero (not important) using a nonparametric chi‐square test.

The diversity of respondents' reliance on the resources of support was illustrated as such: Some respondents did not rely on certain sources of support (score zero). This was illustrated by their scoring of the highest‐rated items in each of the three factors that emerged from the factor analysis, namely for *members, God*, and *therapist*. For those who scored zero for each of these three items, scoring was calculated for how respondents rated each of the other resources.

Paired samples *t*‐tests were conducted to compare the number of f/f meetings before COVID‐19 to the number of virtual meetings and f/f meetings in the past week. Independent samples *t*‐tests were conducted to compare participants who experienced an increase in drug craving to those who did not relative to other items surveyed. All analyses were conducted using the Statistical Package for the Social Sciences, version 26 (SPSS; IBM Corporation).

## RESULTS

There were 347 respondents in the first wave survey who listed methamphetamine as their principal drug of abuse. They are characterized in Table 1. Regarding their patterns of recovery, they reported having their first Twelve Step meeting at age 30.2 (SD = 10.5); 75.8% had completed all Twelve Steps and 98% reported having had a spiritual awakening. The large majority (88.8%) currently had a sponsor and had, themselves, sponsored other NA members (83.6%). Most (64.3%) seldom or never attended traditional services in a house of worship, but they reported that on most days they engaged in NA prayer (85.9%) and felt God's presence in their lives (81.6%).

**Table 1:** Respondents surveyed in the first wave survey (*N* = 347)

	Mean (SD); %
Age	49.7 (11.8)
Ethnicity	
White	85.60%
Black	5.20%
Hispanic/Latino	5.20%
Asian	0.60%
Other	8.10%
NA Craving	
Before COVID	0.65 (1.79)
Last week	0.93 (1.93)
Had a history of intravenous drug use	47.10%
Referred to NA by a healthcare professional	27.00%
Last used substances, years	13.4 (10.5)
Had received nonsubstance psychological treatment	49.60%

Responses for the period during the COVID‐19 pandemic indicated that they attended fewer f/f meetings during the past week (namely, when stay at home was expected) than what they estimated for the period before the onset of COVID (0.83 [SD = 1.73] vs. 3.57 [SD = 2.44], *t* = 19.59, *df* = 342, *p* < .001). Respondents attended significantly fewer f/f meetings during the COVID‐19 period (x̄ = 0.83, SD = 1.73) than they did before the pandemic (x̄ = 3.57, SD = 2.43; *t*[342] = 19.59, *p* < .001, Cohen's *d* = 1.28). Differences in resources scored between the two periods did not meet a small (or greater) size of the effect.

Participants reported higher drug craving during the pandemic than before (0.93 [SD 1.93] vs. 0.65 [SD 1.79], (346) *p* = −3.004, *p* = .003]). Those with higher craving reported having a shorter antecedent length of abstinence from alcohol and other drugs (*t*[147.83] = −3.411, *p* < .001). Those with greater involvement in providing assistance to other members reported less craving than those who did not, as indicated by their sponsoring other members (odds ratio [OR]: 6.05, *p* < .001) and performing NA service (OR: 6.28, *p* < .001).

For the 304 responses to the second wave, Table 2 lists the resources that respondents relied on for support for their recovery and provides results of the factor analysis for the scores recorded by respondents for each of the respective resource choices. The rotated solution revealed a simple structure and explained 57.66% of the total variance. The items were loaded onto three factors, with respective portions of the variance associated with each of them. For identification purposes, the respective factors can be labeled by inspection as 1—social (29%); 2—spiritual (17%); and 3—professional (12%), approximating the type of roles that the factors played in the respondents' recovery process.

Table 2 also provides the results for the non‐parametric chi‐square tests comparing the portion of respondents who indicated a resource was important to some degree (scores 1–4) versus not important (score zero) during the *Before COVID‐19* time period. It includes the mean scores (on the 0‐4 scale) of respondents ascribed to the resources supporting their recovery, all *p* < .001, and the percent of respondents who scored them as important. The majority of resources (9 of the 12) were rated as important to some degree by the large majority of respondents (>90% of those in factors 1 and 2), but the remaining three items were outliers with fewer respondents regarding their relative importance to recovery. Based on these findings, an additional item was created that reflected institutionally sanctioned support; and has a value of 1 if a respondent rated *therapist*, *medication*, or *house of worship* as important, and zero if the respondent rated none of the three items as important. At least one of these three non‐NA supports was rated as important by 75.4% of respondents.

**Table 2:** Factor analysis of 12 resources scored by respondents for the period Before COVID = 19 (*N* = 304)

Resources	Factor			Important^a^	*χ* ^2^ b	Cohen's *h*
	1	2	3			
Other members	0.858			95.4%	**251.0**	2.28
NA literature	0.760	0.372		93.8%	**233.0**	2.13
Face‐to‐face	0.748			93.8%	**233.0**	2.13
My sponsor	0.704	0.324		92.4%	**219.0**	2.02
Service work	0.584	0.437		94.1%	**236.0**	2.16
Spiritual awakening	0.509	0.505		96.1%	**258.0**	2.35
Meditation	0.465	0.322		93.1%	**226.0**	2.08
God		0.811		92.4%	**219.0**	2.02
NA Prayers	0.435	0.617		89.5%	**189.0**	1.82
House of worship		0.445		43.4%	**5.0**	−0.26
Therapist			0.881	60.5%	**13.0**	0.42
Medication			0.713	51.6%	0.0	0.06
Rotation sums of squared loadings	3.44	2.07	1.41	‐	‐	‐
Percentage of total variance	28.69%	17.22%	11.75%	‐	‐	‐
Number of items	6	3	2	‐	‐	‐

*Note*: Significance threshold for *p* = .05; significant *χ* ^2^ are in **bold**.

^a^ important (vs. nonimportant).

^b^ nonparametric *χ* ^2^.

Almost half of the respondents (47.4%) reported having experienced a relapse since joining NA (x̄ = 7.83 [SD = 10.26]) years ago, with a mean duration of 16.66 (SD = 34.4) months. Notably, there was no clinically significant portion on any of the resource items (score 1–4, vs. 0) as to whether or not respondents indicated having relapsed since joining.

Responses on relative reliance on different sources of support are provided in Table 3 for those who scored no support at all (scores zero) versus those who scored any support (1–4) for three respective resources, *members, God*, and *therapist*. This is illustrated by considering the patterns of reliance for respondents who indicate no support (score zero) on the three resources, *other members, God*, and therapist, as listed in Table 3. Thus, respondents who indicated that *other members* were not important to their recovery were no less likely to rate *God* and *spiritual awakening* as resources important to their recovery. Respondents who indicated that *God* was not important to their recovery were more likely to rate social items as important to their recovery; and were least likely to rate *NA prayer* or *house of worship* as important. Respondents who indicated that a *therapist* was not important to their recovery were more likely to rate all other NA‐based items as more important to their recovery, but not medications.

**Table 3:** Respondents who scored the resources *other members, God*, or *therapist* as not important

Items they did rely on	Other members (*n* = 14)		God (*n* = 23)		Therapist (*n* = 120)	
	%	*h*	%	*h*	%	*h*
Other members	—	—	65.2%	0.62	**90.8%**	1.91
Face‐to‐face	0.0%	—	65.2%	0.62	**90.0%**	1.85
NA literature	0.0%	—	56.5%	0.26	**89.2%**	1.80
Sponsor	**14.3%**	−1.59	52.2%	0.09	**88.3%**	1.75
Service work	**21.4%**	−1.22	55.2%	0.21	**90.0%**	1.85
God	42.9%	−0.28	—	—	**89.2%**	1.80
NA prayer	**14.3%**	−1.59	**26.1%**	−1.00	**85.8%**	1.60
Spiritual awakening	42.9%	−0.28	60.9%	0.44	**92.5%**	2.03
Meditation	28.6%	−0.88	69.6%	0.81	**88.3%**	1.75
House of worship	28.6%	−0.88	0.0%	—	**27.5%**	−0.93
Therapist	**21.4%**	−1.22	43.5%	−0.26	—	—
Psych Meds	**14.3%**	−1.59	47.8%	−0.09	**19.2%**	−1.33

*Note*: Resources they did score important (1–4) (*N* = 340). Significance threshold for *p* = .05; percentages for significant nonparametric *χ* ^2^ values are in **bold**.

Differences between genders also indicated a different pattern of reliance. Among male respondents, only 35.9% indicated that professional help (including both therapist and/or medications for psychological problems) was a resource for recovery support whereas 63.2% of females found professional help to be a resource.

## DISCUSSION

Methamphetamine use and misuse represent a major public health problem. In an attempt to address it, a 2005 US Federal Act combatting the “Methamphetamine Epidemic” led to a decline in the availability of the drug, but since 2007, related rates of morbidity and mortality have increased substantially, as evident in reports for poison control centers^11^ and US federal findings.^10^ There are no medications with FDA approval for MUD to aid in treatment, but the NIDA^17^ has recommended that contingency management and cognitive behavioral treatment may be useful. Similar recommendations have been made on the basis of a review of recent peer‐reviewed studies.^21^

Alcoholics Anonymous (AA), a behaviorally oriented approach to alcohol use disorder (AUD), originated in 1935, and a definitive review of literature on AA facilitation showed it more effective than other established treatments, such as cognitive behavioral therapy, for encouraging abstinence.^22^ In relation to nonalcohol use disorders, NA was initially developed by opioid‐dependent persons employing AA's Twelve Step model. Stimulants, including methamphetamine, constitute a major portion of that NA members' experience. In a recent study by NA's World Service Center of 8239 members, 49% listed stimulants as their “main drugs used,” more commonly than opioids (Jane Nickels, email correspondence, May 5, 2020). The survey data we report here indicate that some persons with MUD may be aided in achieving recovery from this drug over the course of membership in NA. Clinicians can turn to FDA‐approved medications for SUDs, such as naltrexone for opioids and AUD. Effective treatment for stimulants such as methamphetamine, however, has neither FDA‐approved pharmacologic options based on empirical study nor are there psychosocial modalities well established for these disorders.^21^

NA, however, is a resource that is widely available, and documentation of recovery for some persons who indicate methamphetamine as their primary drug problem supports the option that it could be useful as a resource for clinicians or clinical programs in stabilizing MUD recovery. NA reports over 33,500 meetings worldwide and could be potentially useful in combination with professional care for MUD. Sources of “recovery capital” are defined as options available to persons with SUD to aid in achieving stable recovery. NA may serve as a source of recovery capital for MUD. The World Health Organization includes in its definition of recovery capital peer‐based and culturally related support for discovering meaning and purpose in life.^23^

Based on our experience with Twelve Step members, we, therefore, selected resources within NA that members could use in bolstering their recovery and solicited respondents to score the degree to which they found these respective resources support their recovery. Resources were scored for two periods, “Currently,” (i.e., during the pandemic during which they were responding) and “before the pandemic,” (i.e., the patterns of reliance, before, and therefore independent of the pandemic). Scoring before the pandemic is reported here to clarify the respondents' patterns of support independent of the COVID‐19 era. We found that the difference in resources did not meet the criteria for a small effect in scores distinguishing the two periods. Effective transition from f/f to virtual meetings during the COVID‐19 period illustrates the adaptability of the Twelve Step format even when the typical mode of meetings was usually not available.

Notably, however, craving ratings were higher for the COVID‐19 period than in the period before the pandemic. Craving was also associated with a shorter period of membership and lesser involvement in the NA format of sponsorship and service to other members. To provide comparable findings for the potential resources of support outside the fellowship, we included three possible options, namely a *house of worship*, an *outside therapist*, and *medications for psychological (nondrug) problems*. Reliance on at least one of these three items was scored by the large majority of respondents. This underlines the need to examine the many non‐NA sources relevant to recovery, indicating that an examination of other community‐based options for support merits examination. Family, friends, and occupational roles are illustrative of this larger pattern of support, and also underline the importance of examining the interaction between the many sources of support outside NA that are available to members and those sources that are in NA. While interactions among these resources are complex, they indicate the importance of considering such resources needed by clinicians for treatment planning in treating a given patient. The finding that 27% of respondents were referred to NA by a health professional also indicates the possible utility of the fellowship for professionals treating MUD patients.

Respective members may draw differently on the resources available to them. This was illustrated by certain members whose scores reflected that they did not rely on three of the resources that were scored highly by other respondents. For example (as in Table 3), the large majority of those who indicated not relying on a therapist did indicate reliance on their sponsor and other members. Additionally, the finding that females are more likely than males to rely on professional help reflects further the differences in the use of resources among members.

## LIMITATIONS

The findings presented here are limited to the small number of persons accessed from the larger NA membership. Certain limitations in this approach are clear. Respondents were fully committed members, having many years since their first encounter with NA, had experienced a spiritual awakening, and had involvement with sponsorship. None of the data we accessed came from NA members who had recently joined or had left the fellowship because of relapse. Only a limited number of resources within or outside the fellowship were presented for scoring. While these are illustrative of respondents' options, other resources are important as well. Additionally, respondents' reports of abstinence as reported here could not be corroborated by independent confirmatory evidence, such as urinalyses.

## CONCLUSION

MUD is a major public health problem, in terms of the high degree of morbidity and mortality associated with it, and the need for sufficient resources for its treatment. This study of NA members who have this disorder and the ratings for sources of support they turn to indicates that at least, for some persons with this disorder, the fellowship can offer a non‐pharmacologic approach to achieving recovery. The findings are also useful in gaining an understanding of how members themselves with MUD see their recovery as stabilized in the fellowship. Such findings can be helpful for investigating mechanisms of action for this peer‐based program, and as a resource for clinicians to consider for their patients with MUD.

## CONFLICT OF INTEREST

The authors declare no conflict of interest.
